# Antidepressant-like Effect of *l*-perillaldehyde in Stress-induced Depression-like Model Mice through Regulation of the Olfactory Nervous System

**DOI:** 10.1093/ecam/nen045

**Published:** 2011-06-22

**Authors:** N. Ito, T. Nagai, T. Oikawa, H. Yamada, T. Hanawa

**Affiliations:** ^1^Oriental Medicine Research Center, The Kitasato Institute, Japan; ^2^Kitasato Institute for Life Sciences and Graduate School of Infection Control Sciences, Kitasato University, Tokyo, Japan

## Abstract

Perillae Herba (a leaf of *Perilla frutescens*) has been prescribed as one of the component herbs in certain Kampo (Japanese herbal) medicines that are used clinically for the improvement of depressive mood. *l*-Perillaldehyde (PAH) is a major component in the essential oil containing in Perillae Herba, but its antidepressant-like effect has not been reported. To clarify the antidepressant-like effect of PAH, the inhaled effect of PAH on stress-induced depression-like model mice prepared by subjection to a combination of forced swimming and chronic mild stresses was investigated. The degree of the depression-like state was measured by the animal's duration of immobility using a forced swimming test. Inhalation of PAH (0.0965 and 0.965 mg/mouse/day, 9 days) significantly shortened the duration of immobility of the depression-like model mice and did not affect locomotor activity. However, another odor substance, cinnamaldehyde containing in Cinnamomi Cortex, exhibited no reduction in the immobility. The reduction in the immobility induced by the inhalation of PAH was prevented on anosmia-induced mice prepared by intranasal irrigation with zinc sulfate. These results suggest that the inhalation of PAH shows antidepressant-like activity through the olfactory nervous function.

## 1. Introduction

Depression is a long-lasting mental disorder, and the prevalence for lifetime estimates with the majority in the range of 8–12% in 10 countries in Europe, the U.S. and Asia [1]. Patients with depression are often prescribed antidepressants, but antidepressants have adverse effects such as dysuria, digestive dysfunction and sexual dysfunction. Therefore, safer and more effective treatments for depression are needed.

In an animal model using a forced swimming test (FST), it was reported that the odor of lemon has an antidepressant-like property [2]. This finding suggests that the sensory action via the olfactory nervous system might be associated with the antidepressant-like effect. Therefore, it is expected that treatments for depression using odor inhalation will be developed. When patients themselves decoct and take Kampo (Japanese herbal) medicines, they can inhale the odor of the decoction, indicating that pharmacological effects of the odor via the olfactory nervous system may be expected.

Perillae Herba is prescribed as one of the component herbs of such Kampo medicines as kososan (Xiang-Su-San in Chinese) and hangekobokuto (Banxia-Houpo-Tang in Chinese), which are used clinically for the improvement of depressive mood and in which antidepressant-like activities have been reported experimentally [3, 4]. It has also been reported that the administration of rosmarinic acid or caffeic acid containing in Perillae Herba exhibited antidepressant-like activities in an animal model of depression [5]. However, the antidepressant-like effect of *l*-perillaldehyde (PAH), which is a major component of essential oil containing in Perillae Herba, has not been reported.

The present study investigated whether the inhalation of PAH shows an antidepressant-like activity by using stress-induced depression-like model mice. The antidepressant-like activity of PAH through the olfactory nervous system was also studied by using anosmia-induced model mice.

## 2. Materials and Methods

### 2.1. Animals

Seven-week-old male ddY mice (Japan SLC, Hamamatsu, Japan), weighing 35–40 g, were used. The mice were housed under conditions of constant temperature (23 ± 2°C), humidity (55 ± 10%) and a 12 h light cycle (lights on at 8:00) with food and water available *ad libitum*. All animal experiments were performed according to the Guide for Care and Use of Laboratory Animals at The Kitasato Institute and Kitasato University.

### 2.2. Drugs and Reagents

**Figure 1:**
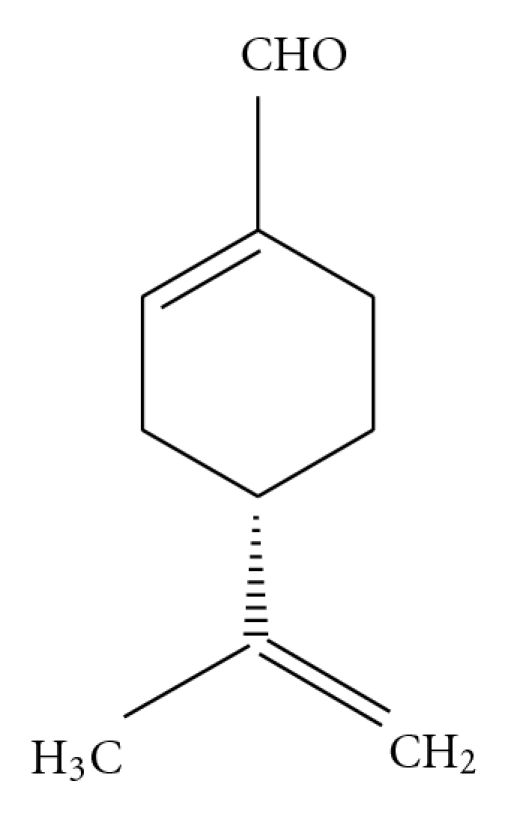
Structure of *l*-perillaldehyde (PAH).

*l*-Perillaldehyde (PAH; Figure 1) (Aldrich Chemical Co. Ltd., St. Louis, MO) was diluted in 0.1, 1.0 and 10% (v/v) with ethanol, and cinnamaldehyde (CAH) (Wako Pure Chemical Industries, Osaka, Japan) was also diluted in 10% (v/v) with ethanol. Toledomin^®^ (milnacipran hydrochloride) (Asahi Kasei Pharma Corp., Tokyo, Japan) was suspended in distilled water. ZnSO_4_ (Wako) was dissolved in 5% (w/v) with saline. Isovaleric acid (Wako) was diluted in 1% (v/v) with water.

### 2.3. Drug Treatment

**Scheme 1:**
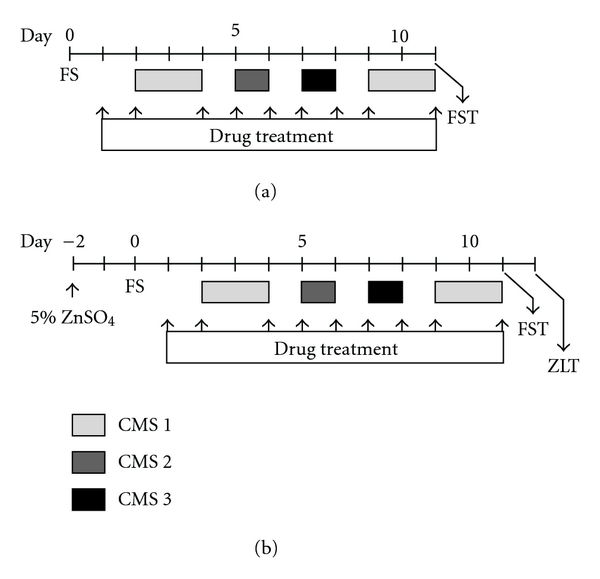
Schedule for the drug treatment on stress-induced depression-like model mice (a) and preparation of anosmia-induced mice (b). (a) Briefly, mice were individually placed into water for 15 min (forced swimming; FS). After 2 days, the mice were exposed to chronic mild stress (CMS 1, CMS 2 and CMS 3), which consisted of three different stress situations. The mice were then placed again into water at 60 min after the final treatment of the drug, and the total duration of immobility during a 5 min forced swimming test (FST) was measured. Drugs were treated at days 1, 2, 4, 5, 6, 7, 8, 9 and 11 (9 times). (b) Briefly, intranasal irrigation with 20 *μ*l of 5% ZnSO_4_ was performed slowly into the bilateral nose under light anesthesia 2 days before FS. After exposure to stresses (mentioned above), the total duration of immobility during a 5 min FST was measured. Following day, the time which the mice spent on the 1% isovaleric acid side was measured for 5 min in the ZnSO_4_-lesioned test (ZLT). Drugs were treated as same schedule as above (9 times).

The mice were made to inhale PAH (0.1, 1.0 and 10%) or CAH (10%) by dropping the solution on an area between the nose and eyes in a volume of 10 *μ*l/mouse once daily at days 1, 2, 4, 5, 6, 7, 8, 9 and 11 (9 times) (Scheme 1(a)). Milnacipran hydrochloride (MIL) (60 mg/kg) was administered orally to the mice by intragastric gavage in a volume of 0.5 ml/mouse once daily at days 1, 2, 4, 5, 6, 7, 8, 9 and 11 (9 times).

### 2.4. Stress-Induced Depression-Like Model Mice

The stress-induced depression-like model mice [3] were prepared by a combination of modified forced swimming (FS) twice with 11-day interval [6, 7] and chronic mild stress (CMS) [8, 9]. Briefly, the mice were individually placed into 5 l glass beakers (height 27 cm, diameter 18 cm) filled with 4   of water (23 ± 1°C) for 15 min. The beakers were separated by non-transparent panels to prevent the mice from seeing each other. After 15 min in the water, the mice were removed and allowed to dry with a drier before being returned to their home cages. The mice were then separated into groups by measuring the duration of immobilities for the first 5 min of FS to minimize the variability of immobility among the groups. A mouse was judged to be immobile when it ceased struggling and remained floating motionless in the water, making only those movements necessary to keep its head above water. After 2 days, the mice were exposed to CMS, which consisted of three different stress situations: tilting of the cage twice 30 degrees from the horizontal (CMS 1), pouring 200 ml of water onto the sawdust bedding of the cage (CMS 2) and shaking the cages at 200 rpm by a Green S. Seriker II (Vision Scientific, Kyunggi, Korea) (CMS 3). These stress situations were applied for 48, 24 and 24 h, respectively, with 24 h intervals (Scheme 1). The mice were then placed again into water at 60 min after the final treatment of the drug, and the total duration of immobility during a 5 min FST was measured.

### 2.5. Spontaneous Locomotor Activity Test

The spontaneous locomotor activities of the mice were evaluated by the open field test. Briefly, the mice were individually placed in the center of an open field (40 × 40 × 20 cm) with the field divided into 25 equal squares (Sanki Kagaku Kogei, Kawasaki, Japan) and allowed to move freely for 15 min. The total number of line crosses for the first 5 min were counted to minimize the variability of the total number of line crosses among the groups. Eleven days later, the mice were again individually placed in the open field and the total numbers of line crosses were counted during a 5 min interval 60 min after the final drug treatment.

### 2.6. Anosmia-Induced Mice

Anosmia was induced according to the modified method of Alberts and Galef [10]. Briefly, intranasal irrigation with 20 *μ*l of 5% ZnSO_4_ was performed slowly into the bilateral nose under light anesthesia with Nembutal (Dainippon Pharmaceutical Co., Ltd, Osaka, Japan) 2 days before FS. The control mice were intranasally perfused with saline (Scheme 1(b)).

### 2.7. ZnSO_4_-Lesioned Test (ZLT)

The degree of anosmia was evaluated by a behavior of escape from a bad smell [11]. Briefly, the mice were individually placed into the center of an emptied cage in which a center line had been drawn. Kimwipe soaked in 1% isovaleric acid solution, which is a bad smell essence, was placed on one side of the line, and kimwipe soaking water on the other side. One day after FST, the time which the mice spent on the 1% isovaleric acid side was measured for 5 min (Scheme 1(b)). If anosmia had been induced in the mice, which meant they showed a low perception of odor, the latency of the 1% isovaleric acid side of the mice was longer than that of the control mice.

### 2.8. Statistical Analysis

All data were expressed as the mean ± standard error of the mean (SEM). Results were analysed by one-way analysis of variance (ANOVA). Post hoc comparisons, if applicable, were carried out using Fisher's PLSD, Dunnett's or Tukey's tests. *P*-values less than  .05 (*P* <  .05) were considered indicative of significance.

## 3. Results

### 3.1. Inhalation of PAH, but Not CAH, Reduces the Immobility of Stress-Induced Depression-Like Model Mice in the FST

**Figure 2:**
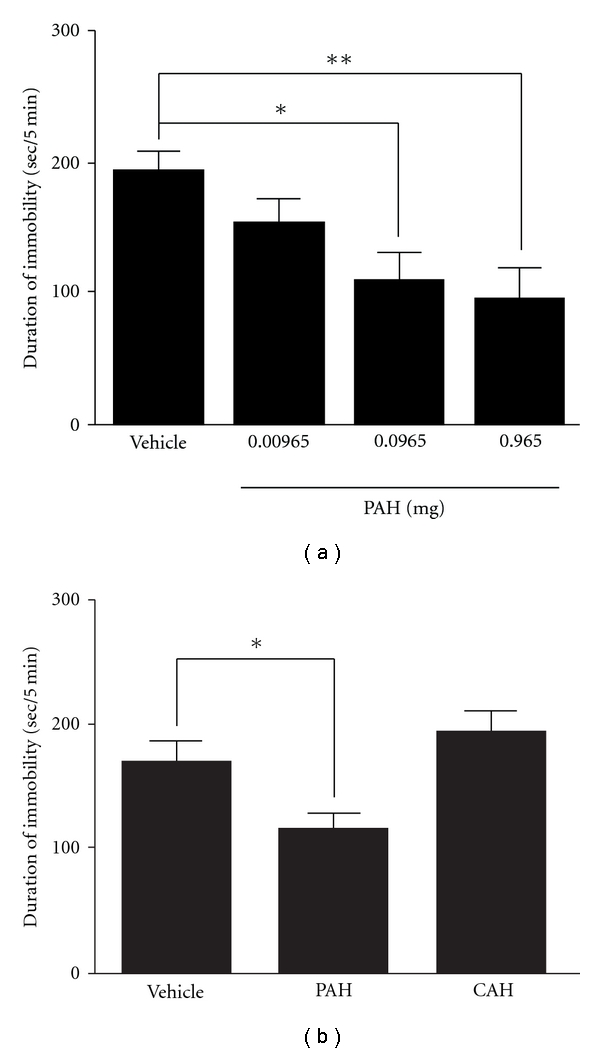
Effects of the inhalation of PAH and/or CAH on the duration of immobility of stress-induced depression-like model mice in the FST. (a) After treatments of PAH (0.00965, 0.0965 and 0.965 mg) for 9 days, the duration of immobility was measured. (b) After treatments of PAH (0.965 mg) and CAH (1.05 mg) for 9 days, the duration of immobility was measured. Each column represents the mean ± SEM of 8–10 mice per group. *P <  .05 and **P <  .01 with Dunnett's test (a) or Fisher's PLSD test (b). CAH, cinnamaldehyde.

Sixty minutes after the last inhalation, PAH (0.0965 and 0.965 mg/mouse/day) significantly reduced the duration of immobility in a dose-dependent manner as compared with the vehicle-treated mice (*P* <  .05 and *P* <  .01, resp.) (Figure 2(a)). However, the inhalation of CAH (1.05 mg/mouse/day) did not reduce the duration of immobility as compared with the vehicle-treated mice (Figure 2(b)).

### 3.2. Inhalation of PAH Does Not Affect the Spontaneous Locomotor Activity of Mice

Sixty minutes after the last inhalation, PAH (0.00965, 0.0965 and 0.965 mg/mouse/day) did not change the total counts (177.6 ± 23.6, 165.8 ± 19.2 and 190.4 ± 19.5, resp.) of line crosses for 5 min as compared with the vehicle-treated mice (173.5 ± 31.9) (data not shown).

### 3.3. Behavioral Verification of Anosmia-Induced Mice

**Figure 3:**
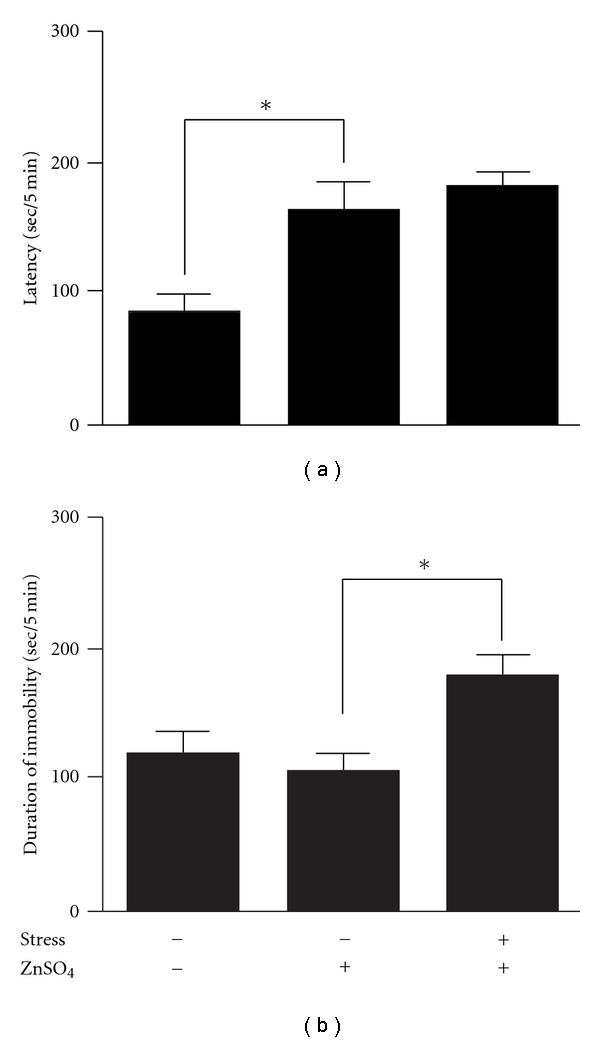
Influences of ZnSO_4_ on the latency of 1% isovaleric acid side in the ZLT (a) and the duration of immobility of stress-induced depression-like model mice in the FST (b). Each column represents the mean ± SEM of 10–11 mice per group. *P <  .01 with Fisher's PLSD test. ZLT, ZnSO_4_-lesioned test.

The latency of the ZnSO_4_-treated mice on the 1% isovaleric acid side was significantly increased compared with the saline-treated mice under the stress-free condition (*P* <  .01); however, the latency of the ZnSO_4_-treated mice was not changed under the stress condition compared with the stress-free condition (Figure 3(a)). The duration of immobility of the ZnSO_4_-treated mice in the FST was significantly increased under the stress condition compared with the stress-free condition; however, the duration of immobility under the stress condition was not changed in the ZnSO_4_-treated mice compared with the saline-treated mice (Figure 3(b)). Moreover, the increase of immobility under the stress condition was shortened by oral administration of an antidepressant, MIL, in the ZnSO_4_-treated mice (data not shown).

### 3.4. Reduction of Immobility due to Inhalation of PAH Is Blocked in Anosmia-Induced Mice

**Figure 4:**
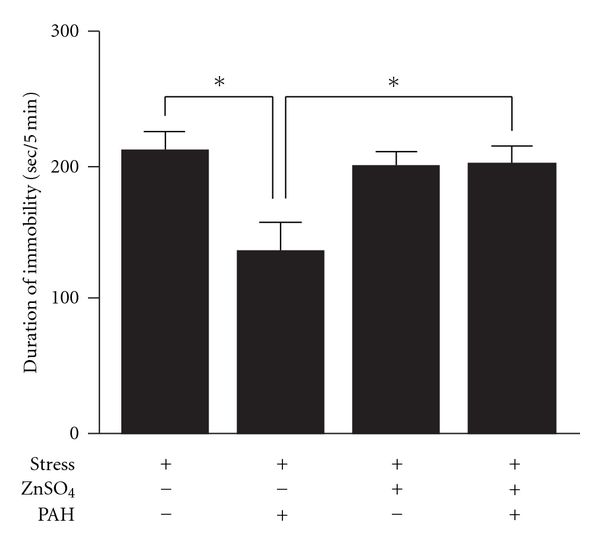
Effects of PAH on the duration of immobility in the FST against anosmia-induced depression-like model mice. After treatments of PAH (0.965 mg) for 9 days, the duration of immobility was measured. Each column represents the mean ± SEM of 10–11 mice per group. *P <  .01 with Tukey's test.

Under the stress condition, the reduction of immobility by the treatment of PAH (0.965 mg/mouse/day) was significantly prevented in the anosmia-induced mice (*P* <  .01; Figure 4).

## 4. Discussion

In the present study, we have shown that the inhalation of PAH containing in Perillae Herba showed an antidepressant-like effect in the stress-induced depression-like model mice, and that its effect was exhibited via the olfactory nervous system.

It is considered difficult to design inhaled doses of PAH both because inhaled drugs are not directly administered into the body and because the sensitivity of the olfactory nervous system varies between humans and rodents. In our study, PAH was inhaled at a dose which was approximately 1.3–130 times lower than the concentration of PAH containing in the water-extract of Perillae Herba (2 g) in kososan, which is used clinically as a one day dosage [12], because rodents have more sensitive olfaction than humans. The inhalation of PAH reduced the immobility in a dose-dependent manner (Figure 2(a)), and did not affect spontaneous locomotor activity (data not shown), indicating that the inhalation of PAH shows an antidepressant-like property. However, CAH, which is a major component of the essential oil containing in Cinnamomi Cortex, did not reduce the immobility in the present study (Figure 2(b)), and it has also been reported that essential oils such as jasmine, ylang-ylang, clove, sandalwood, peppermint, lavender, rose, camphor and pine have no effects on immobility [2]. These results suggest that not all essential oils have an antidepressant-like effect. In the present study, we demonstrated the unprecedented treatment of PAH by dropping it on an area between the nose and eyes in mice. The advantages of this treatment are that each mouse can inhale a constant dose of essential oil, and the mice are not exposed excessively to additional stress against a novel environment, as they are in the method of inhalation in which an apparatus is used for essential oil application [2]. Conversely, since the essential oil was directly dropped onto the area between the nose and eyes, the possibility that the cutaneous absorption of PAH contributed to its antidepressant-like effect cannot be excluded. Therefore, we investigated whether the antidepressant-like effect of PAH is blocked in the mice lesioned olfactory nervous system with the intranasal irrigation of ZnSO_4_. When the degree of anosmia by the treatment of ZnSO_4_ was verified in the behavioral experiment, the ZnSO_4_-treated mice had a significant disruption of the olfactory nervous function (Figure 3(a)). Moreover, histological lesion of the olfactory nervous system was also observed in the ZnSO_4_-treated mice by immunohistochemical study (data not shown). Several studies have reported that many characteristic depressive behaviors (disrupted circadian patterns in sleep and feeding; increased irritability and hyperactivity responses to a novel, stressful environment) observed in olfactory-bulbectomized animals are not detected in ZnSO_4_-treated mice [13–15]. In the present study, the immobility of the ZnSO_4_-treated mice in the FST was not different from that of the vehicle-treated mice (Figure 3(b)). Moreover, the oral administration of MIL (serotonin-noradrenaline reuptake inhibitor) significantly exhibited the reduction of immobility compared with the vehicle-treated mice in the anosmia-induced mice (data not shown). These results indicate that mice in which anosmia is induced by ZnSO_4_ can be used to test behavioral pharmacological strategies for depression. The reduction of immobility induced by the inhalation of PAH in olfactory-unlesioned depression-like model mice was blocked in the anosmia-induced mice. These results suggest that the inhalation of PAH exhibits an antidepressant-like effect via the olfactory nervous system, but not via cutaneous absorption.

The oral administration of PAH also exhibited an antidepressant-like effect in the depression-like model mice, although its effective doses of PAH (24 and 120 mg kg^−1^) were very high compared with those for the inhaled study (data not shown). Although it has been reported that the oral administration of PAH acted on the central nervous system [13], the antidepressant-like activity of PAH has never been reported. This study represents the first report showing the antidepressant-like effect of PAH inhalation. In the present study, it is not, however, known whether PAH in the Perillae Herba contributes to the antidepressant-like activity of Kampo medicines such as kososan and hangekobokuto containing Perillae Herba as one of the component herbs. From the report that citrus fragrance contributes to the reduction of doses of antidepressants in an animal experiment [2], the combination of antidepressants and odors may be useful for exhibiting the antidepressant effects in a synergistic manner. Our finding, as well as the report that the odor of lemon exhibits an antidepressant-like effect [2], together present scientific evidence of the antidepressant-like effect of the odor.

Further investigation of the mechanism of PAH on the antidepressant-like effect is now in progress.
